# Virtual learning environment and its relationship with worker characteristics and occupational accident rates

**DOI:** 10.3389/fpubh.2022.1006918

**Published:** 2022-12-16

**Authors:** Wilder Alfonso Duarte Hernández, LydaCamila Gómez Gómez

**Affiliations:** Corporación Universitaria Minuto de Dios – UNIMINUTO, Bogotá, Colombia

**Keywords:** adult learning, online learning, training, virtual learning, accident, risk management, workplace safety

## Abstract

**Introduction:**

Information and Communication Technologies (ICT) have been employed widely in Occupational Health and Safety Management Systems training programs. However, it is necessary to investigate the influence and workers characteristics to ensure effectiveness. The study presents the relationship between demographic characterization and learning styles with the satisfaction and approval of a training course for teachers through virtual modality and the incidence in accident indicators of an educational institution.

**Methods:**

Analytical and longitudinal study. In 2019, 385 teachers participated in a virtual course on falls prevention. Learning styles were surveyed and records of teachers' entrance and approval of the course were consolidated. The evaluation of the course by teachers was reviewed and the behavior of accident frequency and severity indicators were analyzed comparing 2018 and 2019. To determine significant relationships, Cramer's *V* was applied for learning styles and demographic characteristics with access and course approval. ANOVA was applied for the demographic variables and the evaluation given by teachers to the course. *T*-test was used to compare the average values of the indicators for the period 2018–2019.

**Results and discussion:**

Statistical relationships were found between the predominant learning style and access (*P* < 0.01) and approval (*P* < 0.01). Educational level of the population with access (*P* < 0.05) and course approval (*P* < 0.01). In addition to the age range and the rating given to the methodology applied in the course (*P* < 0.05). No differences were found between the values of the indicators. By identifying significant relationships between learning styles and demographic characteristics of the working population and the use of virtual learning environments, it is important to continue researching the influence of workers' characteristics and didactic methodologies for the design of virtual learning environments that encourage workers to follow safe procedures during their work.

## Introduction

Within the framework of the Occupational Safety and Health Management System (OSHMS), organizations must design and implement an annual training program that provides knowledge for the identification of hazards and control of work-related risks, which is enforced at all levels of the organization (1, 2). A training program seeks to strengthen the formative process within the organization, since one of its purposes is “to improve the current and future employee performance by improving their technical and professional competencies and enriching their specific knowledge, skills, abilities and attitudes” (3).

Bird and Germain (4), for example, state that adequate training promotes an efficient area or department, while contributing to the elimination or reduction of accident or illness risks, among others. Likewise, to meet the needs of a company, they propose that workers must possess certain knowledge, skills and attitudes, thus making systematic training mandatory, which implies a planned process for people to perform their work well.

Currently, trends applied by organizations in their hiring models and the influence of ICTs (5) are aspects to be considered within the management of occupational safety and health within companies, as well as the contemplation of these technological tools within their resources. As mentioned by Serna (6), we live in a world full of changes and uncertainties… the globalization of the economy, the opening of markets, the development of technology and telecommunications revolution are destroying traditional barriers. In this context, organizations will have to continue adapting and improving their management systems through the implementation and/or use of ICTs, while virtual learning environments will become even more important as indispensable tools for the prevention of occupational risks. In addition to the above, the health contingency generated by COVID-19 has forced organizations to rapidly migrate their activities toward a management model based on virtuality.

Although the literature has shown an increase in the description of virtual learning experiences for workers, evidence on its effectiveness and the variables that may influence the learning process needs to be reinforced (7–9).

Ahmadpour et al. (7), in his study, evaluates the attitude toward learning through e-learning for on-the-job training. For this, he considers the variables in three categories: personal factors, environmental factors and technical factors. Correspondingly, he raises some barriers that may affect the learning process under this modality such as: functional capacity of the student, perceived levels of computer literacy, the perception of time dedicated to training activities, and work-based support. Başaga et al. (10) determines the level of occupational safety and health knowledge of employees in the construction sector in Turkey and investigates aspects to consider when delivering trainings. He highlights the interest on the part of workers that occupational safety and health training activities should be practical regardless of age or educational level. His study finds statistically significant relationships between higher age and lower use of the Internet.

On the other hand, the need to identify learning styles when designing virtual learning environments (VLE) for occupational safety and health training processes has also been considered (11–14). In a bibliometric study (11) on the teaching and learning of Ergonomics in virtual and distance education modalities, a minimal proportion of research describing studies on workplace training was found. Some experiences stand out where the training process was successful in workers whose trainers had pedagogical preparation to manage participants according to their learning styles and ensure greater appropriation and reduction of adverse events.

Wolf et al. (14) discuss about the pedagogical strategies applied for the prevention of accidents at work and their low effectiveness, so they analyze strategies that promote active learning styles such as augmented reality. Stuart (13) argues in his study the need to consider this aspect in virtual training processes for workers in teaching.

Rey et al. (12) comment in their literature review on three types of training approach depending on how the student learns: The knowledge-based approach, the demonstration-based approach involving observation and imitation, and the approach based on approximation through practice.

Within the framework of the scenario presented above, in Colombia, entities with the legal obligation to support organizations in their occupational health and safety processes (occupational risk managers) have developed virtual learning spaces in order to contribute to their training programs. One of these courses was applied to teachers at an educational institution that has eight private establishments that provide formal education at the preschool, elementary, and high school levels in different regions of the country, where the need to propose new strategies aimed at achieving an adequate execution and respective evaluation of the annual training program in occupational safety and health became evident. The above situation came about, given that implementing face-to-face training activities for teachers was proving to be ineffective due to the time limitations caused by the constant accompaniment they provide to student groups. Thus, at the end of 2018, the training schedule for the following year included short courses supported by virtual learning environments designed in a generic manner by an occupational risk administrator.

The study describes the relationship between demographic aspects and learning styles with the satisfaction and approval of a training course for teachers through virtual modality about fall prevention, within the framework of the training program, induction and training that every company must develop, according to the guidelines of an OSHMS. Likewise, it is intended to show whether the execution of this course had any influence on the behavior of the organization's accident rates, thus carrying out the verification process in order to comply with the system's objectives.

This research attempts to provide theoretical elements to be considered by those responsible for occupational health and safety management for the assembly of virtual learning environments that support appropriation and teaching processes by workers, taking into account that the health contingency generated by COVID-19 has accelerated the adoption of these technological tools.

## Materials and methods

This was an observational analytical and longitudinal study, with the aim of identifying variables that may influence the development of a course in the virtual modality in the context of the OSHMS training program, through proper monitoring and its influence on the organization's accident rate indicators.

### Sample

The population considered for this study was 385 teachers from eight educational institutions nationwide who were enrolled in a virtual course on fall prevention offered by an Occupational Risk Management Company (ORMC). The inclusion criteria included teachers with an employment relationship of more than 2 months and the exclusion criteria included administrative and support staff.

### Instruments

The Felder and Silverman learning styles test (15), the knowledge evaluation questionnaire applied in the virtual classroom, and the course satisfaction survey were used to collect the information.

The purpose of applying the Felder and Silverman test was to identify the predominant learning styles in the population under study. In Colombia, this model has been applied and, according to Puello and Fernández (16), it has a Cronbach's alpha between 0.7 and 0.9.

The results of the evaluation questionnaire were requested to the ORMC once the course was completed in order to identify whether there was sufficient knowledge appropriation to decide whether to approve the course. Finally, a satisfaction survey was applied in order to know the participants' perception. The participant had to score on a scale of 0 to 5 each of these items:

- The content of the course and its contributions.
- The accuracy of the methodology used.
- The applicability of the knowledge acquired.
- The functionality of the platform used for its development.

To evaluate the influence of the course on the behavior of the accident rate, the accident frequency and severity indicators were monitored during the year of application (2019) and compared with the immediately year prior (2018). The accident frequency index indicates the number of accidents per 100 workers per month, while the severity index indicates the number of days of disability per 100 workers per month (17).

### Procedure

In an initial stage, the research project was socialized to the institution's board of directors, obtaining approval for its development. Taking into account that the main cause of accidents at work was falls, as an intervention strategy for risk control, the institution proceeded in the first semester of 2019 to register its teachers in the virtual course offered by the ORMC for the prevention of slips, trips and falls, while continuing to monitor its accident indicators: the frequency and severity. At the end of the course, participants submitted the course assessment for approval and filled out the satisfaction survey. Subsequently, the institution requested the completion of the learning styles test, socializing the object of the study to the participants and collecting the required informed consents; the instrument was applied virtually to 278 teachers, i.e., 70% of the course participants, regardless of the development or not of the virtual course.

### Statistical analysis

As for the determination of statistically significant relationships, the following measures were considered:

For the identification of demographic aspects and learning styles with classroom entry and course approval, the “Cramer's V” coefficient was used to determine the level or intensity of the relationship between nominal variables, whose value ranges between 0 and 1, with 0 being the absence of correlation between the variables and 1 a perfect correlation (18).

In order to identify the relationship between the evaluation of the perception of satisfaction with the course and demographic aspects, an ANOVA analysis (18) of variance was employed, which made it possible to identify differences in the means or variances of the scores given by the participants on the methodology used, the applicability of the knowledge acquired and the functionality of the platform used for its development with the characteristics of the population.

Finally, to determine the influence of the course developed on the accident rate indicators in the organization, the group contrast *t*-test (18) was used to evaluate whether the differences in the means of the accident frequency and severity indicators for 2018 and 2019 differ significantly from each other.

### Ethical considerations

During the development of the study, the required permissions from the entity were taken into account. The objective of the study and guarantees of the information collected through informed consent were shared with each of the participants. Likewise, the project, with its respective procedures, was endorsed by the research ethics committee of the Corporación Universitaria Minuto de Dios.

## Results

In relation to demographic characteristics, 60% of the population was female, 59% were over 40 years of age and only 20% had postgraduate training. Regarding the predominant learning style in the participating population, it is evident that the active and visual styles were the most frequent with 27.3 and 20.8% respectively (see Table 1). Unfortunately, one third of the teachers did not complete the test.

**Table 1:** Percentage distribution of the population according to demographic characterization and predominant learning style.

**Variable**		**Frequency**	**Percentage**
Sex	Female	232	60.3
	Male	153	39.7
Educational level	NA*	124	32.2
	Primary	4	1.0
	High school	1	0.3
	Technical^a^	3	0.8
	Technologist^a^	8	2.1
	Professional	169	43.9
	Specialist^b^	48	12.5
	Master's degree	28	7.3
Age range	NA*	12	3.1
	18–24 years	13	3.4
	25–30 years	43	11.2
	31–35 years	41	10.6
	36–40 years	49	12.7
	41–45 years	49	12.7
	46–50 years	74	19.2
	51–55 years	51	13.2
	56–60 years	41	10.6
	61–65 years	9	2.3
	66–70 years	3	0.8
Position	Coordinator	7	1.8
	Teacher	378	98.2
Dominant learning style	NA*	124	32.2
	Active	105	27.3
	None	23	6.0
	Visual	80	20.8
	Global	5	1.3
	Intuitive	5	1.3
	Reflexive	3	0.8
	Sequential	12	3.1
	Sensorial	23	6.0
	Verbal	5	1.3

Source: Authors.

^*^Not available.

^a^Formal educational level for technical or labor preparation.

^b^Postgraduate formal educational level of deepening.

Regarding reports of access to the virtual classroom and course approval, 10.6% of the population never enrolled, while 14.5% failed the course (see Table 2).

**Table 2:** Percentage distribution of the population according to platform use and course approval.

**Variable**		**Frequency**	**Percentage**
Use of the platform	Never entered	41	10.6
	Entry	344	89.4
Approval status	Reprobate	56	14.5
	Approved	329	85.5

Source: Authors.

When relating demographic characteristics of the participant population with the records of access to the platform and course approval, statistically significant relationships were found, with a slight strength of association, between the predominant learning style and access to the platform (*P* < 0.01), as well as with course approval (*P* < 0.01). Statistically significant relationships, with a slight strength of association, were also found between educational level with classroom login (*P* > 0.05) and approval of the virtual course (*P* < 0.01) (see Table 3).

**Table 3:** Relationship between demographic characteristics, platform use and approval status.

**Related variables**	**Cramer's *V***	***P***
Predominant learning style/use of the platform	*0.203*	*0.003*
Predominant learning style/approval status	*0.230*	*0.000*
Age range/use of the platform	0.164	0.110
Age range/approval status	0.147	0.215
Educational level/use of the platform	*0.205*	*0.024*
Educational level/approval status	*0.249*	*0.001*
Charge/use of the platform	0.047	0.357
Position/approval status	0.056	0.271

Source: Authors.

Regarding the evaluation of the course by the population and its possible link with demographic variables, there was only a statistically significant relationship between the average rating assigned to the clarity of the methodology used to develop the topics according to age groups (ANOVA: 2.492, *P* < 0.05) (see Table 4).

**Table 4:** Relationship between the evaluation of the course by the participant population and their demographic characteristics.

**Related variables**	**ANOVA *f***	***P***
Knowledge acquired is applicable in their environment/predominant learning style	1.113	0.35
The course met your expectations/predominant learning style	0.446	0.775
The methodology used for the development of the topics was clear/predominant learning style	0.272	0.896
The course content information provided you with knowledge/predominant learning style	0.667	0.616
Content was clear and understood/predominant learning style	0.754	0.556
Concrete answers were provided to concerns/predominant learning style	0.139	0.968
Navigation on the platform was simple and its functionality easy to understand/predominant learning style	1.341	0.255
Concrete answers were provided to concerns/age range	1.365	0.228
Content was clear and understood/age range	0.692	0.656
The course content information provided you with knowledge/age range	1.847	0.090
The methodology used for the development of the topics was clear/age range	*2.492*	*0.023*
Course met your expectations/age range	1.095	0.365
Knowledge acquired is applicable in their environment/age range	1.306	0.254
Navigation on the platform was simple and its functionality easy to understand/age range	0.471	0.830
The knowledge acquired is applicable in their environment/educational level	1.259	0.270
Concrete answers were provided to concerns/educational level	0.266	0.967
The contents were clear and understood/educational level	0.984	0.443
The methodology used for the development of the topics was clear/educational level	0.580	0.772
The course met your expectations/educational level	0.788	0.598
Navigation on the platform was simple and its functionality was easy to understand/educational level	0.459	0.864

Source: Authors.

**Figure 1:**
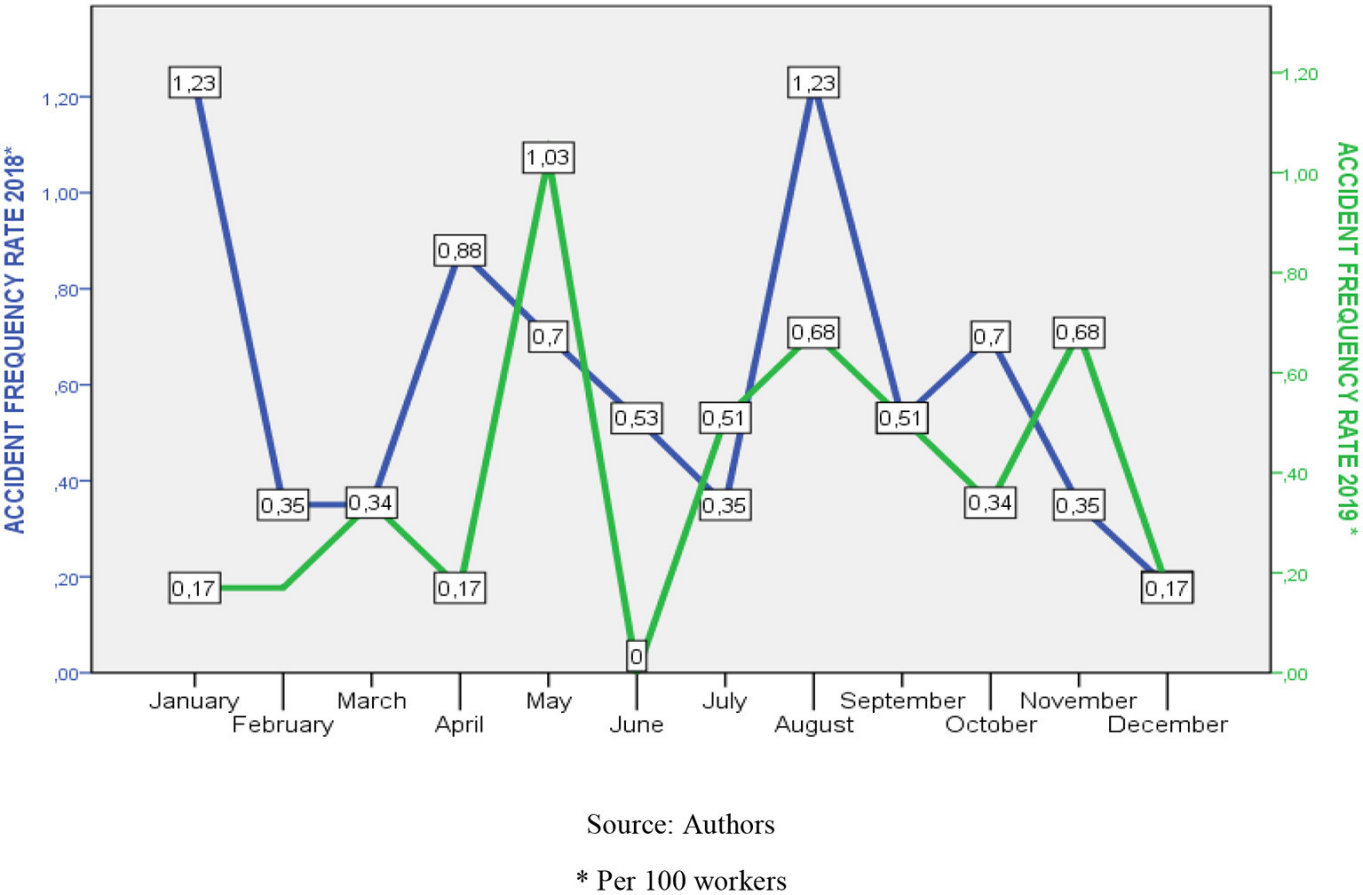
Comparative behavior of the accident frequency rate 2018–2019. Source: Authors. *Per 100 workers.

**Figure 2:**
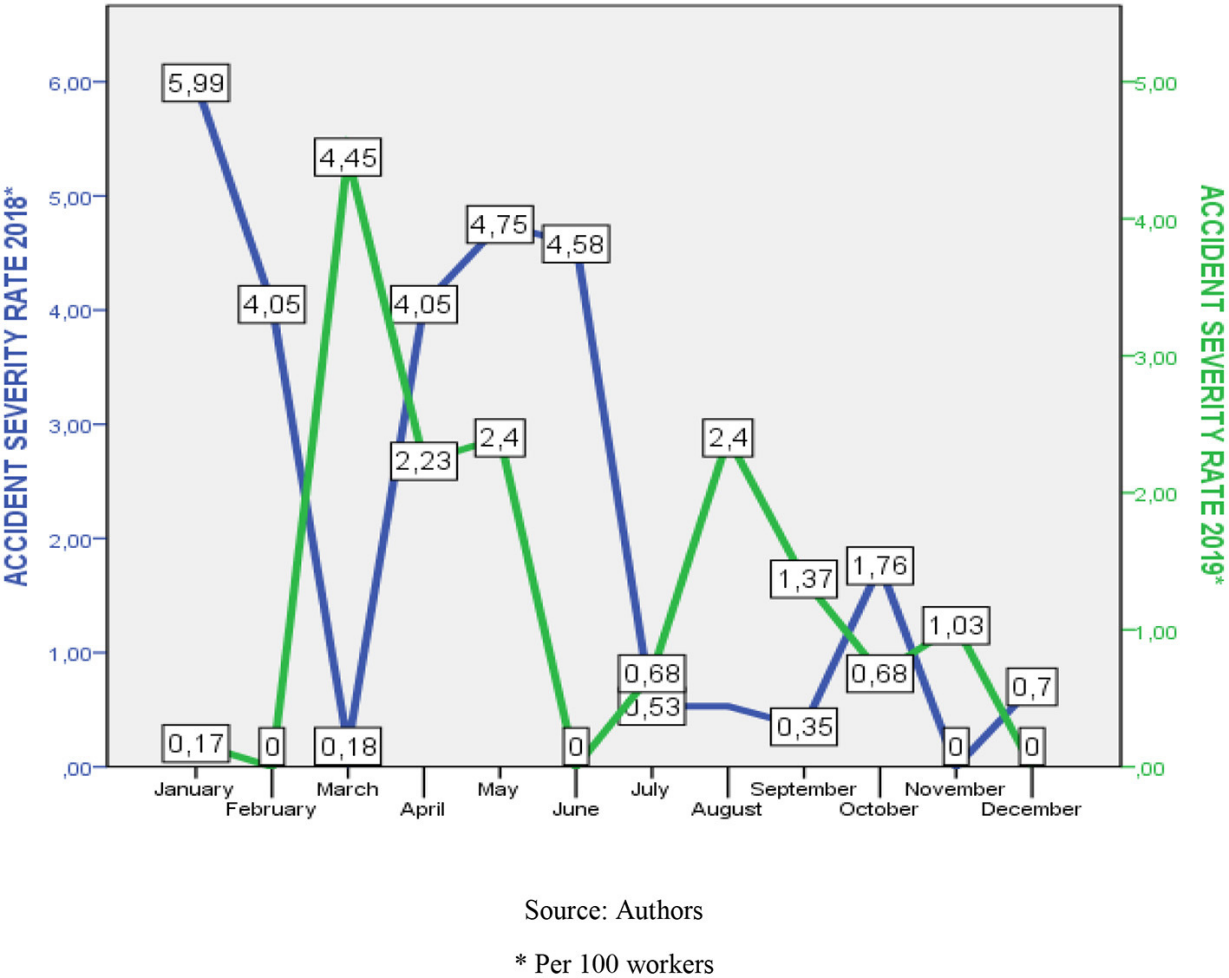
Comparative behavior of the accident severity rate 2018–2019. Source: Authors. *Per 100 workers.

During the application of the course, the accident frequency and severity indexes for the 2019 period were monitored and compared with the 2018 records. Through the *T*-test, the average values of these indexes did not differ significantly (frequency index 2018-2019: 1.74 *P*: >0.05) (severity index: 1.19 *P*: >0.05) (see Table 5, Figures 1, 2).

**Table 5:** Accident rate indicators.

**Indicator**	**Average**		***T*-test**	***P***
Accident frequency rate 2018–2019	2018	0.6150	1.746	0.109
	2019	0.3975		
Accident severity index 2018–2019	2018	2.2892	1.194	0.257
	2019	1.2842		

Source: Authors.

## Discussion

When reviewing the elements implied by the concept of learning styles, the aptitudinal and attitudinal aspects, the programming to receive, memorize and use new information, among others, stand out (15). In the present study, statistically significant relationships were found between entering the virtual classroom and approval the course, with the predominant learning style; the most frequent being visual and active.

According to Felder and Silverman's categories people with a preference for visual learning select visual representations, flow charts, diagrams, schemes and others: they remember better what they see. It is the sensory modality used to perceive information and can be in visual format through charts, graphs, diagrams and/or demonstrations. Whereas people with an active learning domain tend to retain and understand new information better when they do something active with it (discussing it, applying it, explaining it to others). They prefer to learn by rehearsing and working with others (15).

Stuart (13) describes in his study, the theoretical guidelines of Kolb's pedagogical model “Experiential Learning” to be considered in the training process of technicians in the wood furniture industry, for the promotion of safe procedures during the operation of equipment by apprentices. This model argues that learning styles are useful indicators of potential learning success because they provide information about individual differences in learning and information processing. Additionally, it may be considered as one of the most important factors that can influence e-learning and personal academic competence.

Morales and Pereida (19) also suggest the need to examine learning styles and teaching-learning strategies, not only in face-to-face education but also in VLE mediated processes to achieve the desired competencies. This implies the need to examine strategies that encourage student self-management in their formative processes.

On the other hand, although there are studies that question the scarce evidence on the effectiveness of learning based on learning style (20), in the present study significant relationships were found between predominant styles and the approval and entry to the virtual course, which contributes to the relevance of continuing research in this regard and reflecting on the means or strategies to be applied in the virtual spaces to be designed to guarantee the appropriation of learning.

Statistically significant relationships were also found between access and approval of the course according to educational level. Studies have shown that personal factors such as age, educational level and individual interest influence the use of ICTs (10, 21). Zempoalteca et al. (22) identified factors that influence the incorporation of ICTs taking into consideration the self-perception of professors assigned to careers in the area of Administrative Sciences in public higher education institutions in Mexico. They showed that the academic level is relevant because the higher the level, the greater the integration with ICTs, a relationship that was verified through the additional activities that these professors perform, especially with research work or other formal studies they carry out.

Although no statistically significant relationship was found between age and course access and approval, a significant relationship was found with course evaluation, in terms of the clarity of the methodology developed in the course and the average grade score. Ahmadpour et al. (7) found inversely proportional relationships between attitude toward e-learning and age (*P* = 0.01). Which may be attributed to the fact that older people may be accustomed to traditional learning methods and their computer skills are not fully developed. Başaga et al. (10) also find a significant relationship where, as the age of the worker increases, the rate of internet usage decreases drastically.

Finally, no significant changes were found on the indicators of severity and frequency of accident rate when confronting the average values of the 2018 and 2019 periods. The 85.5% of the assigned workers passed the course evaluation questionnaire and only 10.6% of the population never access in the VLE, which was favorable for the achievement of the institution's objectives in terms of providing greater coverage with its OSHMS training program, but it was not effective as it was shown that it had no impact on the accident frequency and severity rates, the ultimate goal of the OSHMS. In their study Chavez and Romero (23) determine the effectiveness of a training course in a Virtual Learning Environment using Kirkpatrick's model finding that when the students' knowledge increased, they felt satisfied, they applied the acquired knowledge and the expected results were achieved, thus concluding that the course was effective. In the present study, through the test of knowledge, it was verified that there was an adequate appropriation of knowledge. Through the test applied, it was determined that the teachers were satisfied with the course (4.7/5); however, as there was no impact on the previously mentioned indicators, it would reflect a poor application of knowledge.

Loosemore and Malouf (24) describe that the knowledge acquired through many training programs is not applied in the workplace. In their study to determine the impact of mandatory training programs on the attitudes of construction workers, they show that these programs generated very little change in the participants' attitudes toward safety. According to statistically significant data, they show only a slight positive change of 3.78% in the mean score of respondents before and after the program. This raises the possibility of evaluating the pedagogical practices applied for the development of training and coaching programs focused on “active training” approaches using didactic strategies such as role-plays, multimedia, excursions, games and other activities to encourage people out of their comfort zone.

Konijn et al. (25) compare differences in the type of training received and the results of awareness and empowerment among Canadian workers who received a training program with active methods such as workshops or under the direction of an instructor and passive means such as online courses or workbooks. Finding a higher degree of awareness and empowerment in groups of workers who received training through a combination of active and passive means (Awareness: OR: 3.86, CI: 2.99-5.01, OR: 2.22, CI: 1.66-2.98, Empowerment: OR: 2.27, CI: 1.83–2.81, OR: 1.70, CI: 1.33–2.17), taking into account variables such as age of the population, place and place of residence, sex, size of the company, exposure to types of hazards and occupational safety and health policies defined in the company.

## Conclusions and recommendations

In the present study, statistically significant relationships were found between demographic variables; such as the educational level and age of the participant population, with the access, approval and perception of the methodology applied in the virtual course on fall prevention. In addition, significant relationships were also found between the predominant learning style of the teaching population and the access and approval of the virtual course.

Thus, the incorporation of learning styles in the design of virtual environments and, therefore, their consequent degree of personalization, continues to be a challenge for the teaching-learning processes and is a relevant object of research. Therefore, it remains to reflect on the importance of updating and considering the demographic characteristics of the working population in occupational safety and health management processes.

However, at the organizational level, studies should delve deeper into the measurement of the effectiveness of training programs, placing greater emphasis on the real impact in terms of knowledge application and results obtained, above the evaluation of knowledge and the level of satisfaction of the participants. Thus, it is advisable to carry out future studies where virtual classrooms are designed considering the intervention of the learning styles of the workers, the demographic characteristics and the didactic methodologies used, for the development of courses or training programs within the framework of the OSHMS.

## Data availability statement

The raw data supporting the conclusions of this article will be made available by the authors, without undue reservation.

## Author contributions

WH: idea conception, information gathering, information processing, consolidation of results, and manuscript construction. LG: idea conception, information gathering, consolidation of results, and manuscript construction. Both authors contributed to the article and approved the submitted version.
